# Pyloric stenosis secondary to peptic ulcer disease in pediatric patients: A case report and review of the literature

**DOI:** 10.1097/MD.0000000000033404

**Published:** 2023-03-24

**Authors:** Jiajia Zhou, Guobin Liu, Xiaofeng Song, Hongjiang Liu, Dengliang Wang, Quan Kang

**Affiliations:** a Department of General Surgery and Trauma Surgery, Children’s Hospital of Chongqing Medical University, National Clinical Research Center for Child Health and Disorders, Ministry of Education Key Laboratory of Child Development and Disorders, China International Science and Technology Cooperation Base of Child Development and Critical Disorders, Chongqing Key Laboratory of Pediatrics, Chongqing, China.

**Keywords:** case report, children, gastric outlet obstruction, peptic ulcer, review

## Abstract

Pediatric pyloric obstruction is a condition characterized by complete or incomplete obstruction of the distal stomach caused by obstructive lesions of the distal stomach, pyloric duct, or proximal duodenum. Congenital hypertrophic pyloric stenosis is the most common cause of pediatric pyloric obstruction, whereas acquired pyloric stenosis is comparatively rare, with peptic ulcer disease being the most common cause.

**Patient concerns:**

We describe a case of a 5-year-old girl who had peptic ulcer disease and developed scarring pyloric stenosis. We also give comprehensive details of the diagnosis and course of treatment.

**Diagnosis:**

Intraoperative findings revealed ulcerative, scarring pyloric obstruction.

**Interventions:**

Conservative treatment failed and surgery was subsequently performed.

**Outcomes:**

No further vomiting symptoms occurred after surgery. And 3 months after surgery, the patient had gained weight on average and had no further complaints.

**Lessons:**

Although scarring pediatric pyloric blockage due to peptic ulcer is less common, emphasis should be placed on rapid diagnosis by accurate gastroscopy, barium meal of the gastrointestinal tract, or ultrasonography. Depending on the patient’s condition, conservative treatment or surgery should be chosen carefully selected.

## 1. Introduction

An obstructive lesion of the proximal duodenum, pyloric duct, or distal stomach may result in pediatric pyloric obstruction. According to Sharma’s classification in 1997,^[1]^ pediatric pyloric obstruction can be caused by congenital intrinsic obstruction of the gastric sinus and pylorus, infantile hypertrophic pyloric stenosis, and acquired causes, with idiopathic hypertrophic pyloric stenosis being the most common.^[2]^ The main causes of acquired pyloric stenosis are scar stenosis due to gastric ulcers, corrosive intake, tumors, polyps, etc. Pediatric pyloric obstruction due to peptic ulcer has been underdiagnosed in the past. In recent years, its incidence has increased because of the extensive use of gastrointestinal endoscopy, but it remains extremely rare.^[3]^ There are only 1 in 100,000 cases from 1967 to date worldwide.^[4]^ Here, we present a case study of scarring pyloric stenosis caused by gastric ulcer, along with comprehensive treatment instructions and related medical documentation for study and reference.

## 2. Case presentation

A 5-year-old girl who had been suffering from repeated vomiting and abdominal pain for >10 days was admitted to our hospital. Prior to her admission, she had been vomiting frequently after drinking cold water for >10 days, averaging 1 to 2 times daily. Primary symptoms included vomiting that occurred 1 to 3 hours after eating, as well as vomiting of stomach contents and vomiting in torrents. Vomiting started out in large amounts and then gradually subsided. Her bloating abdominal pain typically occurred after eating and mainly affected the area around the umbilicus and upper mid abdomen. Vomiting reduced abdominal pain, and loss of appetite also occurred. At the beginning of her illness, anal venting was stopped, but she was able to urinate after using a cork solution, and the pellets in her stool were dark brown.

After the onset of her illness, she was cared for in an outpatient facility with proton pump inhibitors (PPI) and rehydration fluids, but her symptoms persisted. She was admitted to our hospital a day before her first emergence with blood and some small blood clots in her vomit. She had lost 2 kg in weight, had yellow urine with low volume, and had been out of spirits since the onset of the disease.

Regarding her physical condition before this emergency, she was delivered as a full-term and had no delayed meconium. She is physically and mentally mature for her age, but she tends to be very picky about food. She has had no medical problems, surgeries, or gastrointestinal problems in the past. Her parents are both healthy and have no hereditary diseases in their family history.

**Figure 1.**
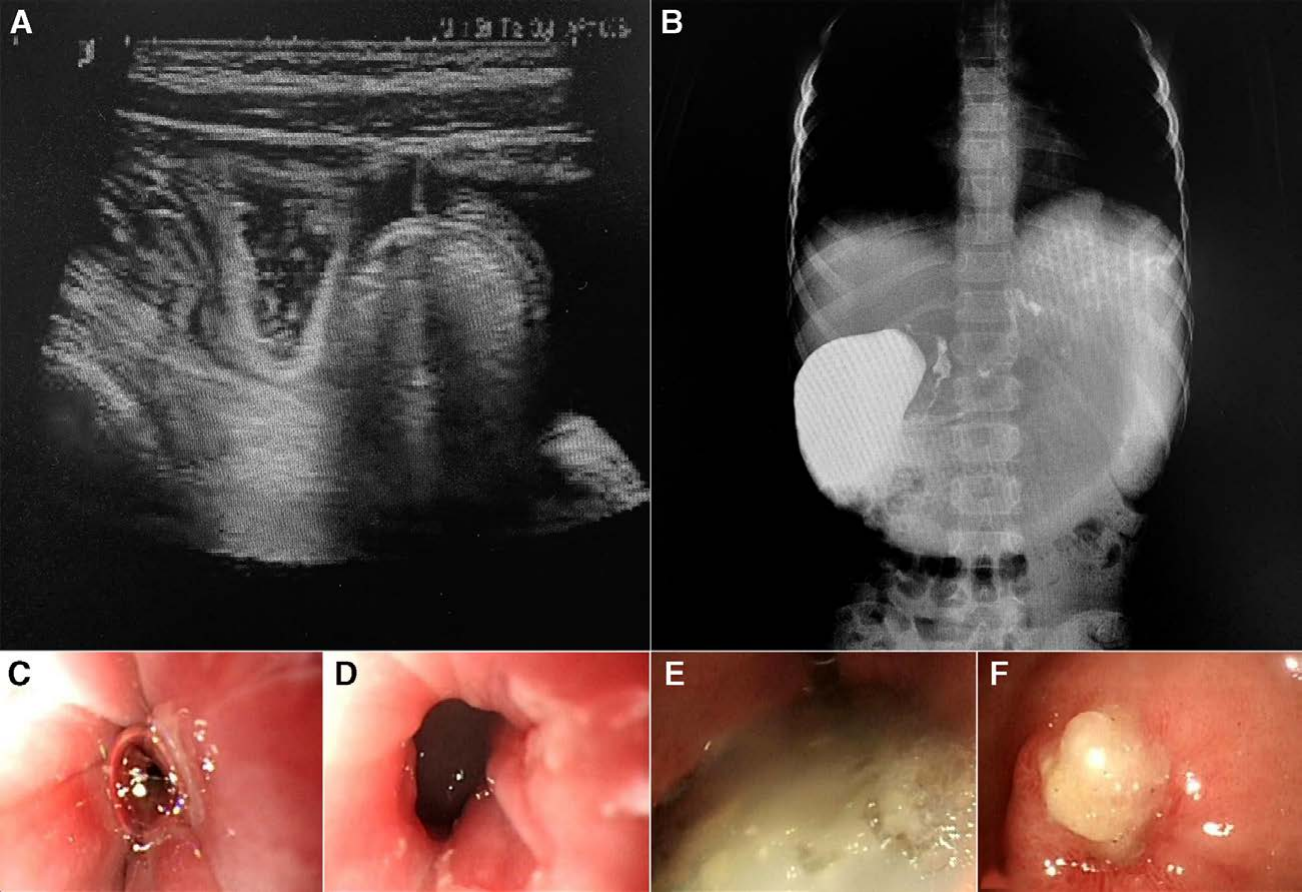
(A) Abdominal ultrasound: more gastric contents were seen in the stomach 30 minutes after eating. (B) Upper gastrointestinal barium meal: the upper gastrointestinal peristalsis was weak, the contrast was difficult to pass through the pylorus, delayed photography, the duodenal circle was poorly displayed, and the proximal jejunum was not visualized. (C–F) Gastroscopy: near the cardia, ulcers covered in white moss are evident. A florid pattern of congestion and edema is present in the stomach sinus. Visible pyloric stenosis is demonstrated with pus-covered ulcers.

After her admission, we inquired about her use of non-steroidal anti-inflammatory drugs (NSAIDs) and found that she had taken a cold medicine shortly before the onset of the illness. However, the specific medications could not be followed up. And we confirmed that she had not taken any corrosive substances. In addition, a physical examination after the child was admitted revealed a poor complexion and mild dehydration. Pressure pain in the upper and middle abdomen and regular bowel sounds were present, but no clear bowel pattern or peristaltic waves were visible. We performed a carbon-13 breath test on her and the presence of *Helicobacter pylori* was not suspected. Ultrasonography was performed (Fig. 1), which was suggestive of gastric retention. Post-meal barium examination (Fig. 1) suggested possible partial pyloric blockage. Further analysis of gastroscopy (Fig. 1) revealed gastric retention, esophageal ulcer, chronic superficial gastritis, and pyloric ulcer with incomplete obstruction. In addition, no *H. pylori* were found in the gastric mucosal tissue collected during gastroscopy. However, we did not know at that time what exactly had caused her gastric ulcer. So we measured the amount of the hormone that produces gastrin in her blood, which was normal, to distinguish it from gastrinoma. According to this evidence, we suspect that her ulcer was caused by her picky eating and snacking habits. Poor eating habits cause more gastric acid to be secreted than consumed, damaging the mucous membrane of the digestive tract and causing ulcers to form. The ulcers bleed when stimulated by suspected NSAID medications. Repeated bleeding and subsequent healing eventually lead to the development of obstruction.

**Figure 2.**
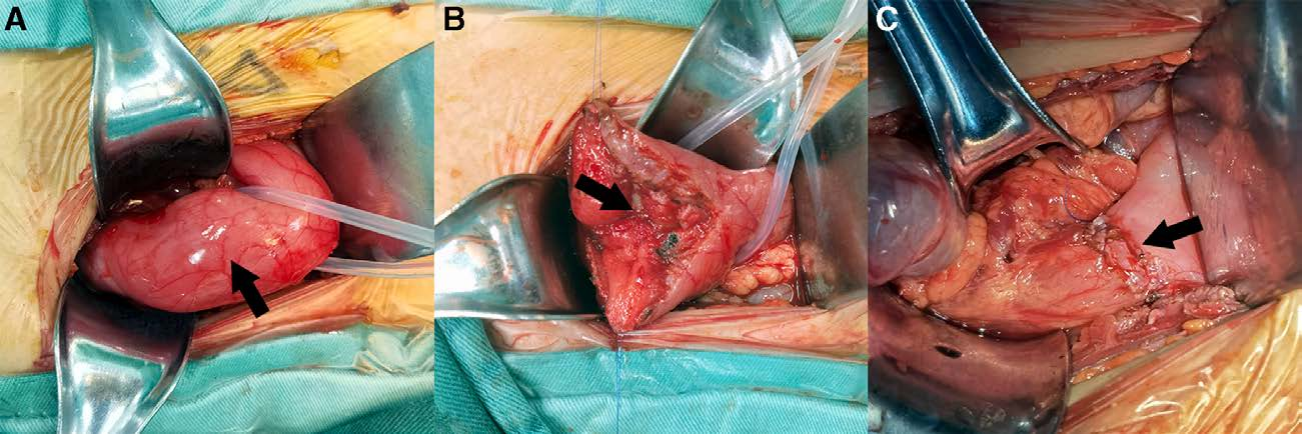
(A) Appearance of the pylorus. (B) Pylorus incision demonstrates local scarring with distinct hyperplasia. (C) After performing gastroduodenal anastomosis.

**Figure 3.**
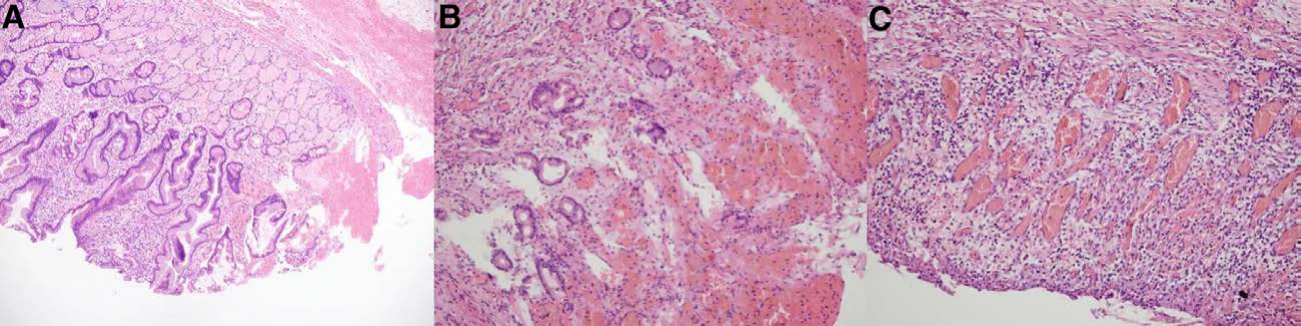
(A) Greater infiltration of single nucleated cells in the lamina propria of the gastric sinus. (B and C) Ulcer development in the pyloric mucosa, deep into the mucosal muscle and submucosa, congested and bleeding, with large acute and chronic inflammatory cell infiltration in the gastric wall.

Based on her clinical presentation and the results of additional testing, she was eventually found to have pyloric stenosis, pyloric portal ulcer, esophageal ulcer, gastric retention, and chronic superficial gastritis. According to gastroscopy findings, the ulcer is active in this child. So, we first tried a conservative approach, which was also in accordance with the family’s wishes. For 2 weeks, we took omeprazole along with a gastric mucosal protector. However, the child’s symptoms improved little to nothing, and the vomiting continued and even seemed to worsen. The parents were very concerned about this and wanted their child’s condition to improve as soon as possible. At this stage, surgery was considered a better treatment for the child’s pyloric blockage. After completing the preoperative tests, we confirmed that she was physically able to tolerate the surgery and then performed the surgery on her. As expected, pyloroplasty should be the first option because it maximally preserves the integrity of the pylorus and restores its function. However, we discovered that the child had significant pyloric scarring during surgery, and traditional pyloroplasty did not seem to have a favorable long-term outcome. Therefore, we placed a gastroduodenal anastomosis (Billroth I) in her. During surgery, it was found intraoperatively (Fig. 2) that the pyloric duct was tough and inflexible, with a length of approximately 4 cm and a diameter of 3.5 cm. There was no obvious abrupt break in the continuity of the pyloric muscle at the end of the duodenum. The mylohyoid layer of the pylorus was transected, and a vesicular ulcer was discovered with no visible active bleeding. Significant local scarring and a small internal diameter were noted. The results of the postoperative pathological biopsy (Fig. 3) showed the development of a pyloric ulcer, mild to moderate chronic inflammation of the gastric sinus, and chronic inflammation of the gastric wall.

After surgery, we treated her with PPI, Cephalosporin antibiotics to prevent postoperative infections, gastrointestinal function control, and rehydration, and the child recovered completely. After postoperative gastrointestinal imaging showed that the anastomosis was open and healed, she was allowed to go home. She gained 4 kg in the first month after surgery and showed no signs of unusual symptoms at the third-month follow-up visit. Her parents were very pleased with the outcome. However, she needs to be more careful with cold medications and give up her selective eating habits. She needs to have another gastroscopy, and the results will show whether she should continue taking PPI medication.

## 3. Discussions

According to studies, pyloric obstruction is a complication in 26.19% of pediatric peptic ulcers.^[5]^ Compared to young girls, boys are more commonly affected.^[6]^ With an incidence of about 7%, they include bulbar ulcers, which are more likely to have bulbar distortion and pyloric obstruction.^[7]^ The condition could be a result of untreated ulcers, repeated attacks, and healing leading to the formation of scar tissue and adhesions with surrounding tissues, ultimately causing persistent pyloric obstruction.^[8]^ According to histology, when a peptic ulcer develops, a significant amount of inflammatory cells are produced, dominated by neutrophils. The necrotic tissue is then replaced by new granulation tissue, which eventually thickens and causes the narrowing of the lumen.

The 2 main causes of peptic ulcers are NSAID use and *H. pylori* infection. Peptic ulcers can also occur as a result of stress, illness, gastrinoma, and unexplained high stomach acid levels. A few more rare causes are autoimmune gastropathy, inflammatory bowel disease, granulomatous gastritis, allergic gastritis, etc. In this case study, the girl was unable to pinpoint the cause of her peptic ulcer. However, we prefer to believe that it was triggered by the ingestion of NSAIDs and the high stomach acid caused by her poor eating habits.

Recurrent vomiting is often the first sign of ulcerative scarring pyloric stenosis in pediatric patients.^[9]^ This may be because the pyloric duct is more narrowed in pediatric patients and is prone to swelling and spasms.^[10]^ Vomiting usually occurs after eating, and the vomit is the stomach contents without bile.^[11]^ Vomiting is the stomach contents without bile, and it typically occurs after eating. Abdominal pain, acid reflux, belching, and other symptoms may occur along with vomiting.^[12]^ Loss of appetite, abdominal pain, bloating, black stools, pale skin, emaciation, abdominal mass, various degrees of anemia, metabolic abnormalities, etc. may also occur in addition to vomiting.^[13]^

The most important methods to confirm the diagnosis of scarring pyloric blockage in pediatric ulcers are gastroscopy and barium meal examination.^[9]^ On gastroscopy, the mucosa of the gastric sinus or pyloric hilus shows obvious ulcerative erosion, even adhesion to pus, thickening of the mucosa around the ulcerated surface, loss of the pyloric duct, and local ulceration.^[12]^ A barium meal is indicated as gastric retention because barium either flows slowly through the pylorus or fails to flow. In addition, ultrasonography has some diagnostic utility. In children with gastric ulcers and pyloric obstruction, edema-like lesions of the gastric fundus are obvious on ultrasonography, and pyloric stenosis may be detected.^[14]^ Postoperative pathologic examination showing persistent inflammatory cell infiltration and hyperplasia of scar tissue in the pyloric mucosa and submucosa may help clarify the diagnosis.^[11]^

The appropriate pharmacological treatment plan, such as an antacid or anti-*H. pylori* agent, can be chosen depending on the initial causes of the peptic ulcers. But in children, especially in late childhood, the degree of pyloric stenosis associated with a long-standing peptic ulcer can be extremely severe. Therefore, in such children, pharmacological treatment alone is not sufficient to relieve the obstruction.^[15,16]^ Because of the local scarring stenosis that causes pyloric obstruction, surgery is required to remove the obstruction.^[17]^ When a child’s condition is complicated by vomiting blood, constipation, perforation, intolerable discomfort, or when conservative medical treatment is unsuccessful, surgery is especially recommended.^[12]^ In addition, some researchers believe that it is a clear surgical indication.^[18]^ Sinus resection, pyloroplasty, gastroduodenal anastomosis (Billroth I), pyloromyotomy, and other surgical procedures are commonly used as treatment options.^[19]^ The most popular surgical option among them is pyloroplasty, which is used to treat benign pyloric obstruction in children.^[20]^ It is also the simplest, safest, and least physically stressful method of treating gastric outlet obstruction^[21]^ and has been shown to be successful in a large number of patients.^[22,23]^ Pyloroplasty can be performed either open or laparoscopically. It has been reported that laparoscopic pyloroplasty slightly increases the risk of mucosal perforation compared with open surgery.^[24]^ In addition, laparoscopic pyloroplasty may carry a higher risk of incomplete dissection of the pyloric muscle compared with open surgery.^[24]^ However, laparoscopic surgery offers the advantages of small incisions, cosmetic results, and minimal invasiveness.^[25]^

In recent years, a growing number of researchers have recommended endoscopic therapy as an alternative to surgery for the initial treatment of ulcerative scarring pyloric obstruction in children^[26]^ and as the initial treatment of pediatric ulcerative pyloric obstruction.^[3]^ Balloon dilatation and endoscopic electrocautery are the main treatment modalities. In some cases, endoscopic therapy has been shown to be better than surgical intervention.^[27]^ Endoscopic balloon dilatation was found to be 95% effective overall in a study of 20 children with ulcerative pyloric stenosis, and the children gained similar weight to children their age during the follow-up period.^[28]^ Therefore, it has been recommended that endoscopic pyloric balloon dilatation be attempted before surgery in children with ulcerative scarred pyloric obstruction,^[29]^ especially in children with peptic ulcers induced by NSAID medication use.^[30]^ However, studies have also shown that the long-term success of endoscopic therapy for ulcerative incisional pyloric stenosis in adolescent patients is questionable. One case reported a failed attempt at endoscopic balloon dilatation.^[4]^ In addition, balloon dilatation treatment must be repeated numerous times, usually over a period of more than 6 to 12 months, which is likely to cause significant psychological damage to the child. In addition, it is difficult to ensure adequate dilatation when there is a risk of bleeding and rupture.^[29,31]^ Children who undergo balloon dilatation more than twice are at higher risk of failure and the need for surgical intervention.^[21]^ In addition, problems such as gastrointestinal perforation have been documented after balloon dilatation, which is a common precursor of pyloric restenosis.^[32]^

Although pediatric pyloric blockage caused by a scarred gastric ulcer is less common, this case reminds us that we should be aware of this etiology and make a quick diagnosis with the help of an accurate gastroscopy, a barium meal of the gastrointestinal tract, or an ultrasound examination. Further studies are needed to determine the efficacy of endoscopic therapy for scarring pyloric blockade in pediatric ulcers. This case also demonstrates the good efficacy of gastroduodenal anastomosis (Billroth I) for scarred pyloric blockade in pediatric ulcers with sustained benefit to the child during long-term follow-up, while conventional surgical technique should still be the mainstay for now. We present a case with a typical clinical presentation, a complete treatment course, and extensive case data in the hope of serving as a reference for identifying and treating pediatric patients with ulcerative scarring pyloric stenosis.

## Acknowledgments

We are grateful to the patient and her parents for participation in this study, as well as for the help of all the physicians in the course of the medical treatment.

## Author contributions

**Conceptualization:** Guobin Liu.

Investigation: Jiajia Zhou.

Resources: Guobin Liu, Xiaofeng Song, Hongjiang Liu, Dengliang Wang.

Supervision: Xiaofeng Song, Quan Kang.

Writing – original draft: Jiajia Zhou.

Writing – review & editing: Jiajia Zhou, Quan Kang.

## References

[1] Sharma KK Agrawal P Toshniwal H. Acquired gastric outlet obstruction during infancy and childhood: a report of five unusual cases. J Pediatr Surg. 1997;32:928–30. 9200104 10.1016/s0022-3468(97)90654-0

[2] Yanmei Z Jinling F. Differential diagnosis and treatment experience of pediatric pyloric obstruction. Kaifeng Med College J. 2000;19:41–2.

[3] Notue YA Mbessoh UI Tientcheu TF. Gastric outlet obstruction secondary to peptic ulcer disease, previously misdiagnosed as idiopathic hypertrophic pyloric stenosis in a 16-year-old girl: a case report. J Surg Case Rep. 2020;7:1–4. 10.1093/jscr/rjaa232 PMC7371009 32704345

[4] Okawada M Okazaki T Takahashi T. Gastric outlet obstruction possibly secondary to ulceration in a 2-year-old girl: a case report. Cases J. 2009;2:8–11. 19123936 10.1186/1757-1626-2-8 PMC2631538

[5] Qiu J. Clinical analysis of 84 cases of peptic ulcer in children. Guide China Med. 2013;11:508–9.

[6] Yen J Kong M. Gastric outlet obstruction in pediatric patients. Chang Gung Med J. 2006;29:401–5. 17051838

[7] Wan-peng L. Analysis of clinical diagnosis and treatment of peptic ulcer in children. Mod J Integr Tradit Chin West Med. 2011;20:2549–50.

[8] Chun Y. Clinical observation of 24 cases of pediatric peptic ulcer with pyloric obstruction. J Med Inf. 2015;7:240.

[9] Bin CC Yu YJ Yong CZ. Pyloric canal ulcer in 36 children. Chin J Pract Pediatr. 2010;25:295–6.

[10] Lei L. Clinical analysis of 25 cases of pyloric duct ulcers in children. Guide China Med. 2013;11:390–1.

[11] Ya-ling X Xiao-yu Z Zhao-yang L. Clinical analysis of 17 cases of pyloric obstruction complicated with infantile peptic ulcet. J Clin Pediatr Surg. 2004;3:419–21.

[12] Xianyong L Jiaxiang W Xinjian C. Surgical treatment strategies for non-hypertrophic pyloric obstruction. Chin J Pediatr Surg. 2013;34:741–5.

[13] Wenqing L. Clinical analysis of 22 cases of pyloric stenosis in pediatric patients. J Pract Med. 2008;24:1971–3.

[14] Jie X Jiayinaerguli-Zhumabieke Xueni Z. Clinical observation on peptic uleer complicated with pyloric obstruction in children. China Contin Med Educ. 2017;9:119–121.

[15] Azarow K Kim P Shandling B. A 45-year experience with surgical treatment of peptic ulcer disease in children. J Pediatr Surg. 1996;31:750–3. 8783092 10.1016/s0022-3468(96)90122-0

[16] Patel RA Baker SS Sayej WN. Two cases of helicobacter pylori-negative gastric outlet obstruction in children. Case Rep Gastrointes. 2011;2011:1–3. 10.1155/2011/749850 PMC3350168 22606426

[17] Ming G Rulin W. A case of pediatric chronic ulcer of the gastric sinus with scarring pyloric obstruction. Anhui Med J. 1993;14:38.

[18] Xue Q. A case of scarring pyloric obstruction with superior mesenteric artery syndrome. Chin J Gen Surg. 2010;25:696.

[19] Boybeyi O Karnak I Ekinci S. Late-onset hypertrophic pyloric stenosis: definition of diagnostic criteria and algorithm for the management. J Pediatr Surg. 2010;45:1777–83. 20850620 10.1016/j.jpedsurg.2010.04.014

[20] Pathak M Saxena R Patel H. Primary acquired cicatrizing gastric outlet obstruction in children. J Indian Assoc Pediatr Surg. 2022;27:38–41. 35261512 10.4103/jiaps.JIAPS_249_20 PMC8853601

[21] Ceccanti S Mele E Frediani S. Laparoscopic pyloroplasty for idiopathic non-hypertrophic pyloric stenosis in a child. J Pediatr Surg. 2012;47:1955–8. 23084216 10.1016/j.jpedsurg.2012.08.009

[22] Feng J Gu W Li M. Rare causes of gastric outlet obstruction in children. Pediatr Surg Int. 2005;21:635–40. 16041609 10.1007/s00383-005-1472-z

[23] Sharma KK Ranka P Goyal P. Gastric outlet obstruction in children: an overview with report of “Jodhpur disease” and Sharma’s classification. J Pediatr Surg. 2008;43:1891–7. 18926227 10.1016/j.jpedsurg.2008.07.001

[24] Jia WQ Tian JH Yang KH. Open versus laparoscopic pyloromyotomy for pyloric stenosis: a meta-analysis of randomized controlled trials. Eur J Pediatr Surg. 2011;21:77–81. 20957601 10.1055/s-0030-1261926

[25] Suolin L Zengwen Y Yingchao L. Laparoscopic gastrojejunal suture anastomosis for pediatric pyloric obstruction. Chin J Pediatr Surg. 2006;27:671–2.

[26] Chan KL Saing H. Balloon catheter dilatation of peptic pyloric stenosis in children. J Pediatr Gastr Nutr. 1994;18:465–8. 10.1097/00005176-199405000-00011 7915308

[27] Chao H. Update on endoscopic management of gastric outlet obstruction in children. World J Gastro Endos. 2016;8:635. 10.4253/wjge.v8.i18.635 PMC5067470 27803770

[28] Pei-yu C Wen-zji O Si-tang G. Investigate on the curative effect of treatment of children’s ulcerative pyloric stricture by endoscopic balloon dilatation. J Clin Pediatr. 2008;26:850–2.

[29] Temiz A Oguzkurt P Ezer SS. Management of pyloric stricture in children: endoscopic balloon dilatation and surgery. Surg Endosc. 2012;26:1903–8. 22234589 10.1007/s00464-011-2124-0

[30] Öztan MO Güngör-Takeş G Çağan-Appak Y. Management of NSAID-related pyloric obstruction in a child using endoscopic balloon dilatation: a case report. Turk J Pediatr. 2018;60:765–8. 31365220 10.24953/turkjped.2018.06.024

[31] Karagiozoglou-Lampoudi T Agakidis CH Chryssostomidou S. Conservative management of caustic substance ingestion in a pediatric department setting, short-term and long-term outcome. Dis Esophagus. 2011;24:86–91. 20659141 10.1111/j.1442-2050.2010.01097.x

[32] You Z Jing A. Analysis of the efficacy of seven cases of pyloric obstruction caused by chemical burns in children. Chin J Pediatr Surg. 2013;34:953–4.

